# A case of extracapsular silicone gel implant rupture with contralateral gel migration

**DOI:** 10.1080/23320885.2024.2357121

**Published:** 2024-05-23

**Authors:** Rebecca Patrick, Sydney Bormann, Heather Karu

**Affiliations:** a School of Medicine and Dentistry, University of Rochester, Rochester, NY, USA; b Sanford School of Medicine, University of SD, Sioux Falls, SD, USA; c Sanford Health Department of Plastic and Reconstructive Surgery, Sioux Falls, SD, USA

**Keywords:** Implant rupture, implant tunneling, symmastia

## Abstract

Symmastia is a rare complication of augmentation mammaplasty that occurs when a breast implant crosses the midline and connects with the contralateral implant pocket. We present a case of implant rupture, migration to the contralateral breast, and ultimate symmastia following a traumatic fall in a patient with prior breast augmentation.

## Introduction

Breast implant rupture is a known complication following breast augmentation. Rupture may occur due to various causes including normal aging of the implant, damage from surgical instruments, or traumatic force to the chest [1,2]. Although trauma-induced rupture is less common with modern breast prostheses, closed capsulotomy, seat belt contusion injury, blunt trauma and compression during mammographic imaging remain causes of traumatic rupture [1,3]. Implant rupture may present with capsular contracture, breast lumps, decreased breast volume and changes in breast shape or firmness [1,2]. Implant rupture may be detected through physical examination, ultrasound or MRI but often remains clinically undetectable. Ruptured implants are usually treated with implant removal, capsulectomy and subsequent implant replacement. Here, we present a perplexing case of implant rupture complicated by migration to the contralateral breast with ultimate symmastia following a fall from horseback.

## Case report

**Figure 1.**
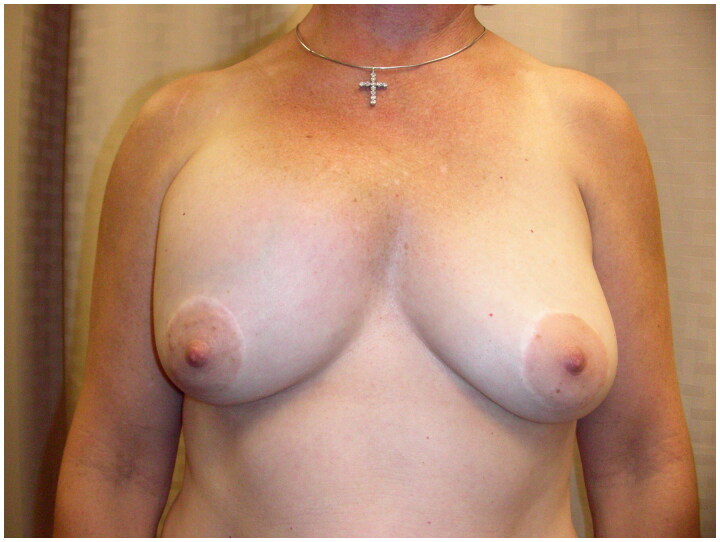
Preoperative image of patient presenting with asymmetry and countour deformity of the right breast.

A perimenopausal woman presented with a change in breast contour of her right breast implant. The right breast was shifted superomedially resulting in asymmetry; however, signs of symmastia were not apparent (Figure 1). She had a bilateral augmentation mammoplasty with silicone implants approximately 20 years prior and no other history of breast surgery. The patient reported placement of a 280 cc implant on the left and a 310 cc implant on the right. She had no significant medical or family history and medications included varenicline and naproxen as needed. Her social history was significant for an extensive history of manual labor in her work as a horse-trainer and a recent increase in upper body exercise. The patient reported smoking approximately one pack per day prior to presentation and drinking an average of one alcoholic beverage per day. The patient reported a fall from horseback with the horse landing on her chest correlated with a change in breast contour. She presented with concerns of swelling above her right breast. A mammogram obtained after symptom onset demonstrated bilateral subpectoral silicone implants without evidence of silicone extravasation with the right implant positioned higher than the left. On examination, a Baker Grade-III capsular contracture of the right breast implant was noted, with the implant noted to be displaced superiorly and medially, extending past the midline. The left breast implant was not palpable. A periareolar scar was noted bilaterally, consistent with her prior augmentation surgery. No nipple discharge or palpable masses were noted. Operative intervention was recommended and bilateral implant removal, capsulectomies and implant replacement were planned. Smoking cessation for six weeks prior to surgery was discussed with the patient and she agreed with the plan.

**Figure 2.**
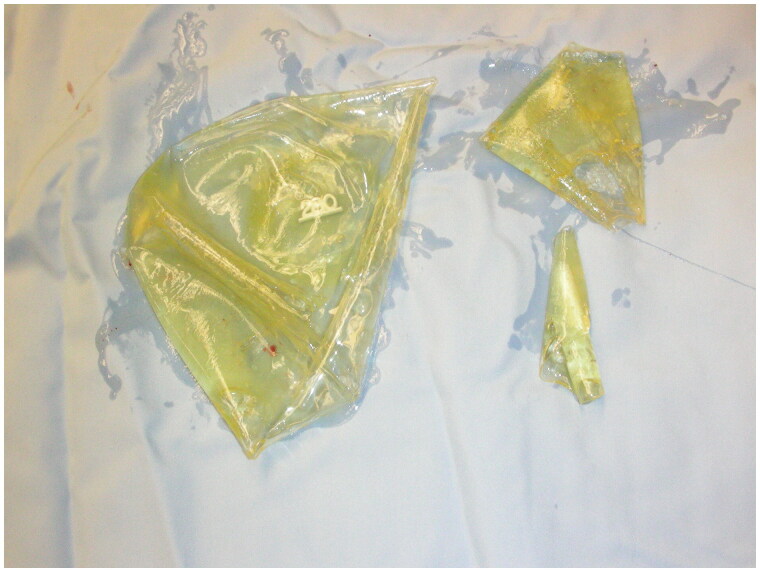
Fragments of left 280 cc breast implant that extruded from the right breast implant space upon entry.

**Figure 3.**
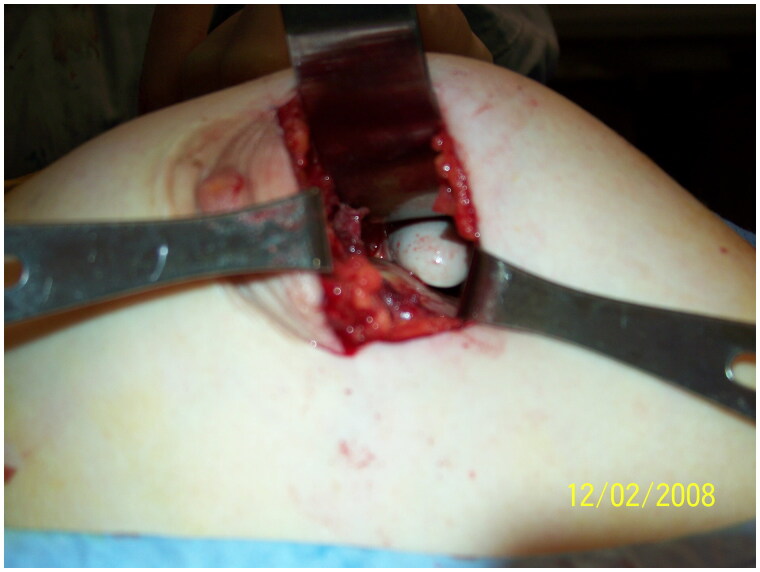
Tunneling between the left and right breast implant spaces as demonstrated by surgeon’s index finger crossing the midline.

Intraoperatively, a periareolar incision was made on the right side and dissection to the submuscular plane was achieved. Upon entering the submuscular space, but before incising the capsule, silicone material flowed freely from the area. The capsule space was incised and an entire, intact 310 cc right implant was removed. After removing the right implant on the right side, silicone continued to leak from the area and fragments of the 280 cc left implant were expressed from the same space (Figure 2). Applying pressure to the left breast caused further expulsion of 280 cc implant fragments and silicone material from the right side. Upon entry into the capsule space on the left side through a similar periareolar incision, no implant was found. A tunnel was visualized connecting the left and right implant spaces (Figure 3). Only a small amount of pooling silicone material was noted. All areas including the tunneling portion were thoroughly cleaned and washed out and bilateral capsulectomies were performed to the degree that was possible. A capsulectomy was also performed on the tunneling space at both entrance points using 3-0 Vicryl sutures to close the tunnel. New high-profile, silicone implants were placed bilaterally in the submuscular space.

## Discussion

The majority of trauma does not cause rupture of breast implants although several case reports following blunt trauma are noted in the literature [1,4–6]. Reported rates of implant rupture vary based on implant manufacturer and patient evaluation. Studies estimate that implants are durable for 6–8 years, after which the rate of rupture begins to increase [2,7,8]. Although there is no standard lifespan estimate for breast prosthesis, the physician estimate ranges from 12–35 years [1]. This patient’s implants were over 20 years old; therefore, implant age was a likely risk factor for rupture. The mammogram obtained after the onset of this patient’s symptoms demonstrated a right implant positioned higher than the left; however, no evidence of silicone extravasation was found. Mammography has a reported sensitivity of 11–69% for the detection of silicone implant rupture, and extracapsular rupture is more often identified with mammography than intracapsular rupture. Intracapsular rupture often requires ultrasound or MRI for identification, and MRI is considered the most accurate imaging modality [9]. In 2022, the Food and Drug Administration published updated recommendations of breast implant screening guidelines. The initial screening imaging examination with either MRI or ultrasound is recommended at five years postimplantation with further imaging every two to three years thereafter [10]. There is no role for diagnostic mammography for implant evaluation in asymptomatic patients with silicone implants; however, patients with breast implants should follow standard breast cancer screening protocols with regular mammograms [11].

Symmastia is a rare complication of augmentation mammaplasty that occurs when a breast implant crosses the midline and connects with the contralateral implant pocket, causing distortion of the vertical intermammary sulcus [12,13]. This phenomenon most commonly occurs in patients with large implants, patients who have undergone multiple augmentation procedures, and those with chest wall deformities. The underlying mechanism of symmastia is a disruption of the medial pectoralis attachments along the sternal border. It is corrected by capsulorrhapy, capsular flaps, acellular dermal matrix, explantation with delayed re-implantation, or a pocket change [13]. A recent report cites iatrogenic injury due to overaggressive tissue dissection and fat grafting using a sharp cannula as potential causes of symmastia [14]. In this case, the defect connecting the implant spaces was repaired and new implants were placed in the same submuscular plane. In future cases, an alernative approach could be considered by placing new implants in the prepectoral space. This may be beneficial in protecting the muscle repair and preventing symmastia recurrence.

Although the age of this patient’s implants likely predisposed her to implant rupture, it does not explain the etiology of the tunneling and resultant symmastia. Perhaps this patient had a predisposition to symmastia following iatrogenic injury to the medial pectoralis border during augmentation 20 years prior. Perhaps this patient had a subtle underlying chest wall deformity or poor tissue integrity predisposing to medial pectoralis border weakness. Perhaps cumulative microtraumas to the medial chest wall throughout this patients’ lifetime with her extensive history of manual labor all contributed to the weakening of the medial pectoralis border. When considering the timing of the fall from the horse and reported onset of symmastia, blunt trauma appears to be the major inciting factor and perhaps a major contributor to implant tunneling and resultant symmastia. Further collaboration with surgical centers to conduct larger studies analyzing environmental factors in patients who develop symmastia following breast augmentation are necessary to determine any formal association.

## Conclusion

Blunt trauma may be associated with implant tunneling to the contralateral implant space and resulting symmastia in patients with prior breast augmentation.

## Ethical statement

Exempted from the IRB approval.

## Patient consent

Written and verbal informed consent was obtained from the studied patient.
